# A bibliometric and visual analysis of colorectal cancer liver metastasis research from 2015 to 2025

**DOI:** 10.1007/s12672-026-04664-3

**Published:** 2026-02-25

**Authors:** Xingxing Xiao, Xiaohe Sun, Jin Sun, Haibo Cheng, Jing Shu

**Affiliations:** 1 Yueyang Hospital of Integrated Traditional Chinese and Western Medicine, Shanghai University of Traditional Chinese Medicine, Shanghai, People’s Republic of China; 2 Jiangsu Collaborative Innovation Center of TCM Prevention and Treatment of Tumor, The First Clinical Medical College, Nanjing University of Chinese Medicine, Nanjing, Jiangsu People’s Republic of China; 3 Jiangsu Province Hospital of Chinese Medicine, Affiliated Hospital of Nanjing University of Chinese Medicine, Nanjing, Jiangsu People’s Republic of China; 4 Shanghai University of Traditional Chinese Medicine, Shanghai, People’s Republic of China

**Keywords:** Colorectal cancer, Liver metastasis, Bibliometric analysis, Research trend, Hotspots

## Abstract

**Objective:**

Colorectal cancer liver metastasis (CCLM) is a common and serious complication of colorectal cancer and is one of the main causes of patient death. This study examines the trend of CCLM research using bibliometric and visualization analysis to provide a reference for further research.

**Methods:**

We searched for publications related to CCLM published between 2015 and March 2025 in the Web of Science and PubMed databases and visualized and analyzed data using R (version 4.4.2), VOSviewer (version 1.6.20), and CiteSpace (version 6.3.R1) software.

**Results:**

A total of 8656 relevant publications were included. The number of CCLM publications has shown a steady upward trend from 2015 to 2025, with articles being the predominant type. The field has developed a larger number of core authors, with Pawlik, Timothy M. scholar having the highest cumulative number of publications. “Cancers” contributed the most publications. China is the country contributing the most publications in the field, but American scholars have the highest average number of citations per publicaton. “colorectal cancer”, “survival”, “cancer”, “chemotherapy”, “liver metastases”, “hepatic resection”, “surgery” and so on are the keywords that appear with high frequency in this field. The most frequently cited journal is “Annals of Surgery”. The most cited publication is “Clinical score for predicting recurrence after hepatic resection for metastatic colorectal cancer—Analysis of 1001 consecutive cases”. The citing journals are mainly in the fields of oncology, surgery, and imaging, while the cited journals also include some journals in other basic disciplines. Clinical research on CCLM has mainly focused on prospective studies, with an emphasis on gender, age, treatment outcomes, and disease-free survival.

**Conclusion:**

This study comprehensively analyzes the current research status and hotspots in the field of CCLM, which can provide valuable references for future research and help other scholars grasp the dynamics of research in this field and discover potential research directions.

## Introduction

Colorectal cancer (CRC) is one of the most prevalent malignant tumors within the digestive system. According to global cancer burden data from 2022, CRC ranks third in incidence among malignant tumors worldwide and second in mortality rates [1]. CRC commonly metastasizes to the liver, lungs, peritoneum, and lymph nodes through direct infiltration, hematogenous dissemination, and lymphatic spread, with liver metastasis being the most frequent occurrence [2]. Colorectal cancer liver metastasis (CCLM) occurs in the advanced stage (Stage IV), and patients may have clinical symptoms such as right upper abdominal pain, jaundice, and hepatomegaly. Its early symptoms are often inconspicuous, and the disease progresses rapidly, with a poor prognosis and significant treatment challenges, which is one of the main causes of patient mortality [3]. Research shows that about 20% to 25% of patients have distant metastasis at initial medical consultation, while another 20% to 25% of patients will develop liver metastasis during disease progression [4, 5].

Meanwhile, clinical management of CCLM has evolved from primarily surgical approaches to multimodal strategies, with systemic therapies increasingly playing a pivotal role. Systemic chemotherapy regimens such as folinic acid, fluorouracil, and oxaliplatin (FOLFOX) and folinic acid, fluorouracil, and irinotecan (FOLFIRI) are now widely employed [6]. The integration of targeted therapies targeting vascular endothelial growth factor (VEGF) and epidermal growth factor receptor (EGFR) has further improved outcomes [7, 8]. Moreover, immunotherapy, particularly immune checkpoint inhibitor treatment for patients with mismatch repair deficiency (dMMR) or high microsatellite instability (MSI-H) metastatic colorectal cancer, has revolutionized the management of this patient subgroup [9, 10]. Understanding clinical treatment and research advances is crucial for improving the management and prognosis of CCLM patients.

Therefore, a comprehensive understanding of the pathogenesis, therapeutic strategies, and prognostic factors of CCLM is essential for the prognosis and treatment of patients with advanced CRC. An in-depth analysis of the research literature in the field of CCLM can help grasp research dynamics, identify potential research directions, and provide guidance for future research and clinical practice. However, given the extensive literature in this field, it is difficult to comprehensively understand and assess this research field by manually searching and reading the literature [11]. Bibliometric studies offer effective approaches to comprehensively and systematically analyze the research field’s characteristics through quantitative literature analysis, revealing the research development patterns and research hotspots [12–14]. To our knowledge, there has not been a comprehensive bibliometric analysis assessing the research status, hotspots, and trends in the field of CCLM. Therefore, we conducted a bibliometric and visualization analysis to elucidate the research status and trends in CCLM to facilitate further research [15, 16].

Bibliometrics is a research method that analyzes and visualizes the research status and trends in a certain field by quantitatively analyzing information such as authors, countries, organizations, journals, keywords, and references [17–19]. In addition, bibliometrics is able to conduct network analysis and visualize the results, helping to reveal connections between studies [20]. There are a number of tools that can be used for bibliometric analysis, such as VOSviewer and Citespace, which can be used to create visual knowledge graphs [21].

In this study, we used bibliometric methods to analyze the knowledge graph of articles and reviews written in English related to CCLM between 2015 and March 2025, revealing the current status of research and development trends in the field, with a view to informing further scientific research and clinical practice in the field of CCLM.

## Materials and methods

### Data source and search strategy

This bibliometric study analyzed publications related to CCLM from January 1, 2015, to March 22, 2025 (incomplete year, for reference only). We conducted a systematic literature search in two major databases: Web of Science Core Collection [WoSCC, including Science Citation Index Expanded (SCIE) to ensure comprehensiveness and accuracy] and PubMed. WoSCC is a well-recognized, high-quality database widely used in scientific research, and PubMed covers an extensive range of biomedical literature, ensuring comprehensive retrieval [22–24]. The search strategy combined subject headings and free-text terms to maximize sensitivity and specificity. Publication language was limited to English, and the type of literature was limited to articles and reviews.

We applied the subject term search method as the search strategy and used the following search formula to perform a literature search in WoSCC: TS=((colorectal* OR colon* OR rectum* OR rectal*) AND (neoplasia* OR carcinoma* OR tumor* OR tumour* OR oncology*) AND (liver* OR hepatic*) AND (metastasis* OR metastases* OR metastatic* OR metastasia* OR metastasize*)) OR TS=(CRLM).

In addition, we entered “Colorectal cancer” in the MeSH Database of PubMed and obtained the following subject terms: “Colorectal Neoplasm”, “Neoplasm, Colorectal”, “Colorectal Tumors”, “Colorectal Tumor”, “Tumor, Colorectal”, “Tumors, Colorectal”, “Neoplasms, Colorectal”, “Colorectal Cancer”, “Cancer, Colorectal”, “Cancers, Colorectal”, “Colorectal Cancers”, “Colorectal Carcinoma”, “Carcinoma, Colorectal”, “Carcinomas, Colorectal”, “Colorectal Carcinomas”. Similarly, when entering “liver metastasis”, no items were found. Therefore, we applied a combination of subject terms and free terms as the search strategy and used the following search formula to retrieve literature in PubMed: (((((((((((((((((Colorectal Neoplasm) OR (Neoplasm, Colorectal)) OR (Colorectal Tumors)) OR (Colorectal Tumor)) OR (Tumor, Colorectal)) OR (Tumors, Colorectal)) OR (Neoplasms, Colorectal)) OR (Colorectal Cancer)) OR (Cancer, Colorectal)) OR (Cancers, Colorectal)) OR (Colorectal Cancers)) OR (Colorectal Carcinoma)) OR (Carcinoma, Colorectal)) OR (Carcinomas, Colorectal)) OR (Colorectal Carcinomas)) AND ((((Liver metastases) OR (Liver metastasis)) OR (Hepatic metastases)) OR (Hepatic metastasis))) OR ((Metastatic liver cancer) OR (Metastatic colorectal cancer))) AND ((“2015/01/01“[Date—Publication] : “2025/03/22“[Date—Publication])), and selected “Clinical Trial” to filter relevant literature.

### Literature screening and data processing

Two independent researchers (XX Xiao and XH Sun) performed the literature screening in three stages: title, abstract, and full-text reviews to identify studies relevant to CCLM. Inclusion criteria required articles and reviews focused on CCLM; conference abstracts, letters, editorials, and non-English publications were excluded. Discrepancies were resolved by discussion, and when consensus was not reached, a third researcher (J Sun) adjudicated. The final datasets were exported as plain text files containing full bibliographic information (title, authors, year, journal, keywords, citations, abstracts, references).

The filtered data from WoSCC were exported in plain text format for data cleansing and duplicate removal in CiteSpace, followed by bibliometric analysis. All retrieved records from both databases were imported into EndNote software for duplicate removal, followed by clinical progression analysis.

### Bibliometric analysis and visualization

Bibliometric and scientific mapping analyses were performed using three software tools. R (version 4.4.2) was used for statistical analysis and visualization of publication trends, types, and distributions. VOSviewer (version 1.6.20) was applied to construct and visualize co-authorship networks, keyword co-occurrence maps, country collaborations, and co-citation networks of journals and literature. CiteSpace (version 6.3.R1) was utilized for burst keyword detection, timeline clustering of keywords, and visualization of citation networks to reveal emerging trends and dynamic changes in research foci.

## Results

### Bibliometric analysis

#### Overview of publication status

**Fig. 1:**
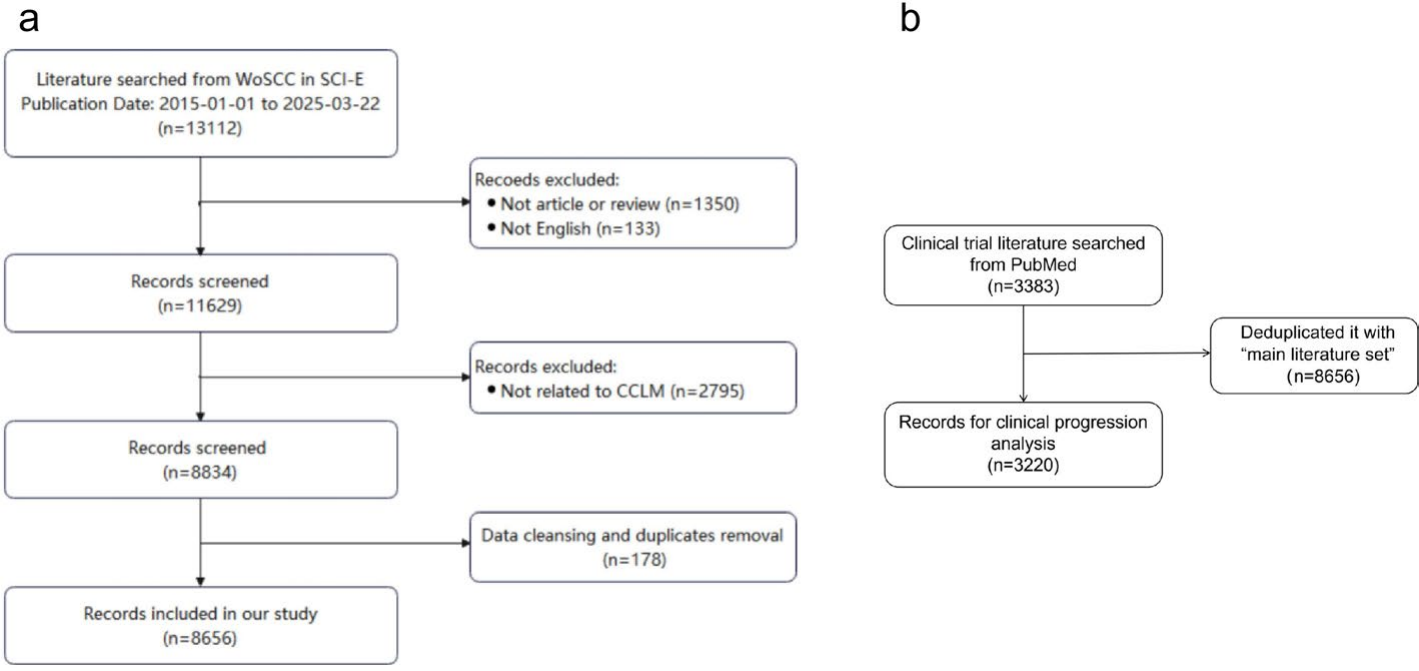
The flowchart of the literature screening

As shown in Fig. 1, a total of 13,112 records published between January 1, 2015 and March 22, 2025 (incomplete year, for reference only), were identified. 1350 records were excluded because the type of literature was not articles or reviews (e.g., conference abstracts, letters, and ongoing papers), and 133 records were excluded because they were written in languages other than English. The remaining 11,629 records were further evaluated by abstract or full-text reading, and 2795 records were excluded as unrelated to the topic of CCLM (the prevention, diagnosis, treatment, or prognosis of CCLM). The filtered data were exported in plain text format for data cleansing and duplicate removal in CiteSpace, and 178 records were excluded. Finally, 8656 studies that met the inclusion criteria and did not meet the exclusion criteria were included in this bibliometric analysis.

After importing them into EndNote and automatically deduplicating based on title, author, and DOI with the 8656 articles previously screened from WoSCC, and manually checking the potential duplicates identified by the software, a total of 3220 studies were obtained for clinical progress analysis.

The 8656 publications used in this study were written by 44,435 authors from 8075 organizations in 108 countries, published in 1014 journals, and cited 191,859 references from 12,108 journals.

#### Annual number of publications and percentage of publication types

**Fig. 2:**
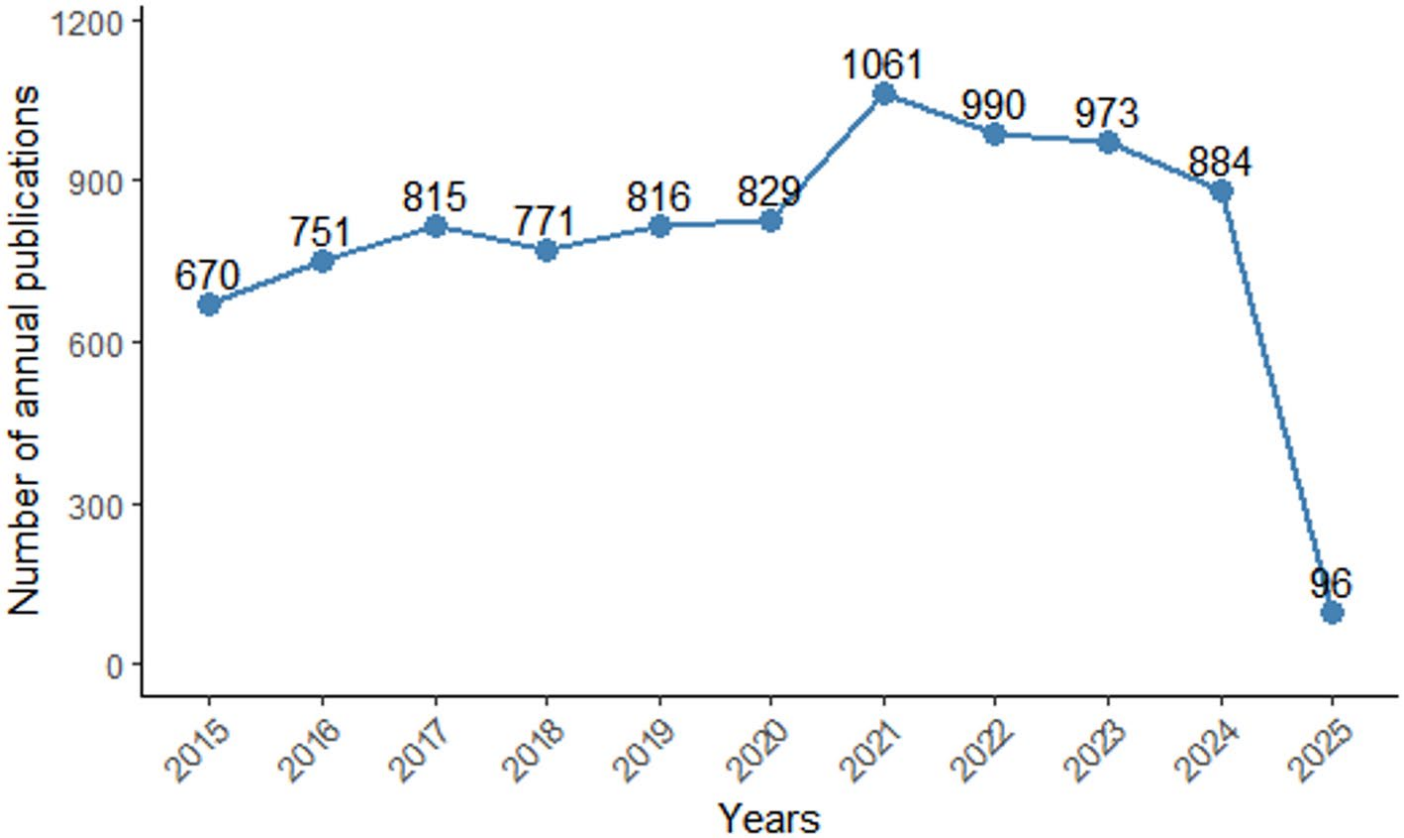
Annual number of publications (The 2025 is incomplete year, for reference only)

We used R (version 4.4.2) to map the annual number of publications and publication shares in the field of CCLM from 2015 to March 2025 (incomplete year, for reference only). Figure 2 shows the temporal distribution of publications in the field of CCLM research. On the whole, the annual number of publications in this field in the past ten years has been relatively stable but still shows a slow upward trend. In 2021, the number of publications increased rapidly, reaching more than 1000, which indicates that this research field has received more and more attention from scholars in recent years and has become mature. After removing other types of publications, such as conference abstracts, editorial material, book chapters, and letters, a total of 8656 publications, comprising 7032 articles (83%) and 1391 reviews (17%), related to CCLM were included in this bibliometric analysis.

#### Bibliometric analysis of authors

**Fig. 3:**
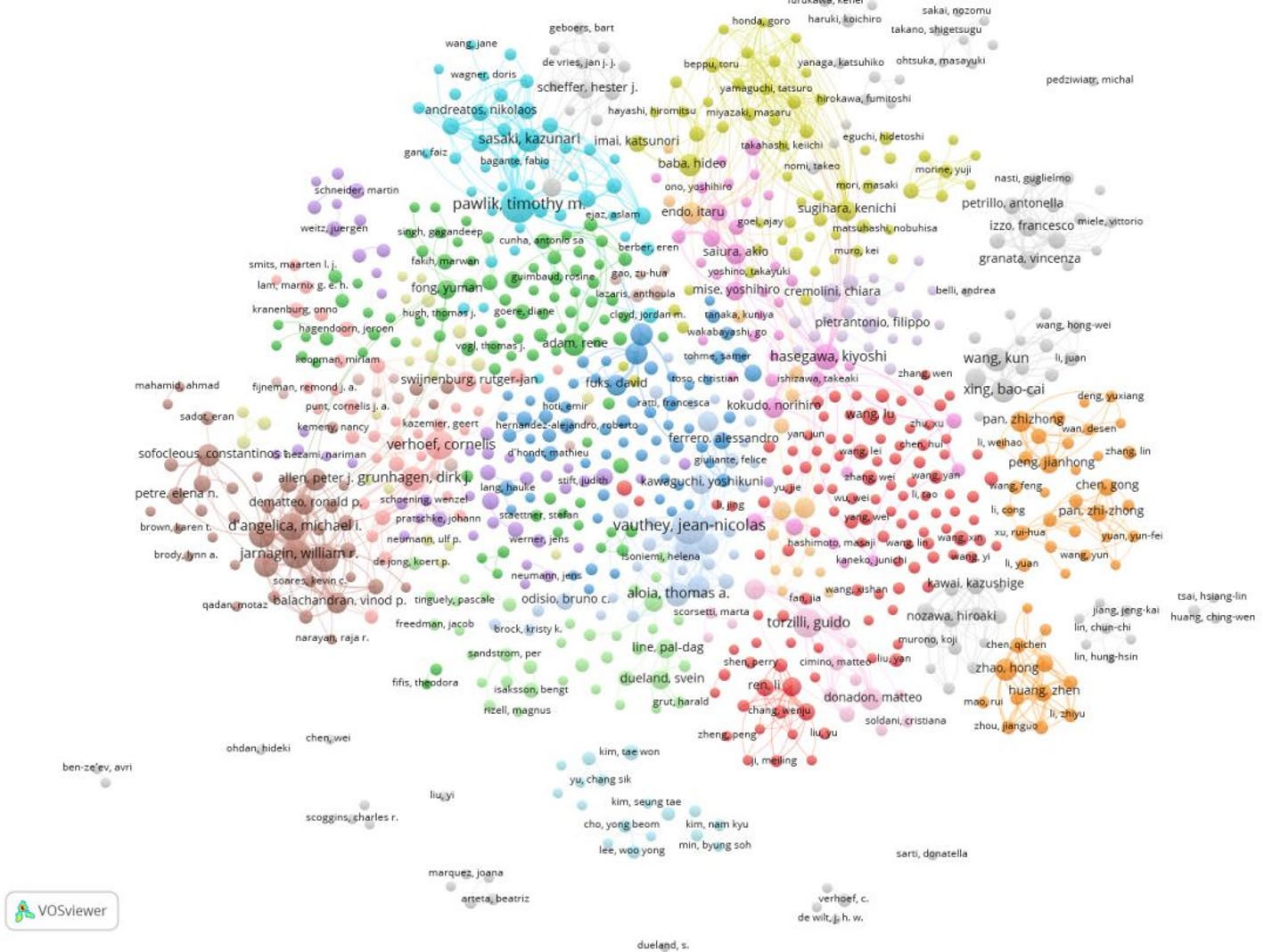
The co-occurrence of authors

The authors of the literature were analyzed to learn about the representative scholars and core research strengths in the field of study [25]. A total of 44,435 authors have contributed to research in the field of CCLM. Using VOSviewer, we constructed the co-occurrence network knowledge graph of authors. A total of 44,435 nodes representing 44,435 authors are shown in the co-occurrence network graph (Fig. 3). Node size is positively correlated with the number of publications by the authors, and links represent collaborations between authors, with Pawlik having the highest cumulative number of publications, followed by Vauthey and Jarnagin, suggesting that they have made notable contributions to the field of CCLM.

The eminent scholar Price [26] stated that half of the papers on the same topic are written by a group of highly productive authors, and this set of authors is quantitatively approximately equal to the square root of the total number of authors, Price’s Law. According to Price’s Law, the threshold for core authors in CCLM was set at a minimum of 8 publications (including 8). Therefore, 869 authors were identified as core authors in the field [27]. Table 1 demonstrates the top nine core authors in CCLM, ranked by their number of publications, and shows their respective citation counts and citations per publication. The highest number of publications, Pawlik, has published a total of 94 publications from 2015 to March 2025 and has received 3168 citations, with an average citation of 33.70 citations per publication.

**Table 1:** Most important authors in the field of CCLM

Rank	Author	Number of publications	Citations	Citations per publication
1	Pawlik, Timothy M.	94	3168	33.70
2	Vauthey, Jean-nicolas	89	3069	34.48
3	Jarnagin, William R.	67	2433	36.31
4	Verhoef, Cornelis	67	1801	26.88
5	Torzilli, Guido	64	1756	27.44
6	Kingham, T. Peter	60	1977	32.95
7	D’angelica, Michael I.	58	2575	44.40
8	Gonen, Mithat	53	2234	42.15
9	Hasegawa, Kiyoshi	51	784	15.37

#### Bibliometric analysis of journals

An analysis of the journals revealed that a total of 1014 journals published articles and reviews related to CCLM in the last decade, with most journals belonging to the fields of oncology and surgery. Table 2 demonstrates information about the top 10 journals by the number of publications, highlighting the most prominent journals in the field. “Cancers” contributed the most publications (302), followed by “Annals of Surgical Oncology” (239) and “Frontiers in Oncology” (187). Regarding journal citations, “Oncotarget” had the highest citations per publication (33.83), indicating high-quality research and significant attention in the field of CCLM.

**Table 2:** Top 10 journals in the field of CCLM

Rank	Source	Number of publications	Citations	Citations per publication	IF [5]	Quartile in category
1	Cancers	302	3567	11.81	4.8	2
2	Annals of Surgical Oncology	239	4733	19.80	3.6	1
3	Frontiers in Oncology	187	1848	9.88	3.8	2
4	EJSO	171	2987	17.47	3.3	1
5	Journal of Surgical Oncology	162	2526	15.59	2.2	2
6	HPB	158	2454	15.53	3.0	1
7	Oncotarget	136	4601	33.83	5.3	1
8	Anticancer Research	131	1133	8.65	1.8	4
9	BMC Cancer	116	3016	26.00	3.7	2
10	Journal of Gastrointestinal Surgery	107	1715	16.03	2.8	1

#### Bibliometric analysis of countries

**Fig. 4:**
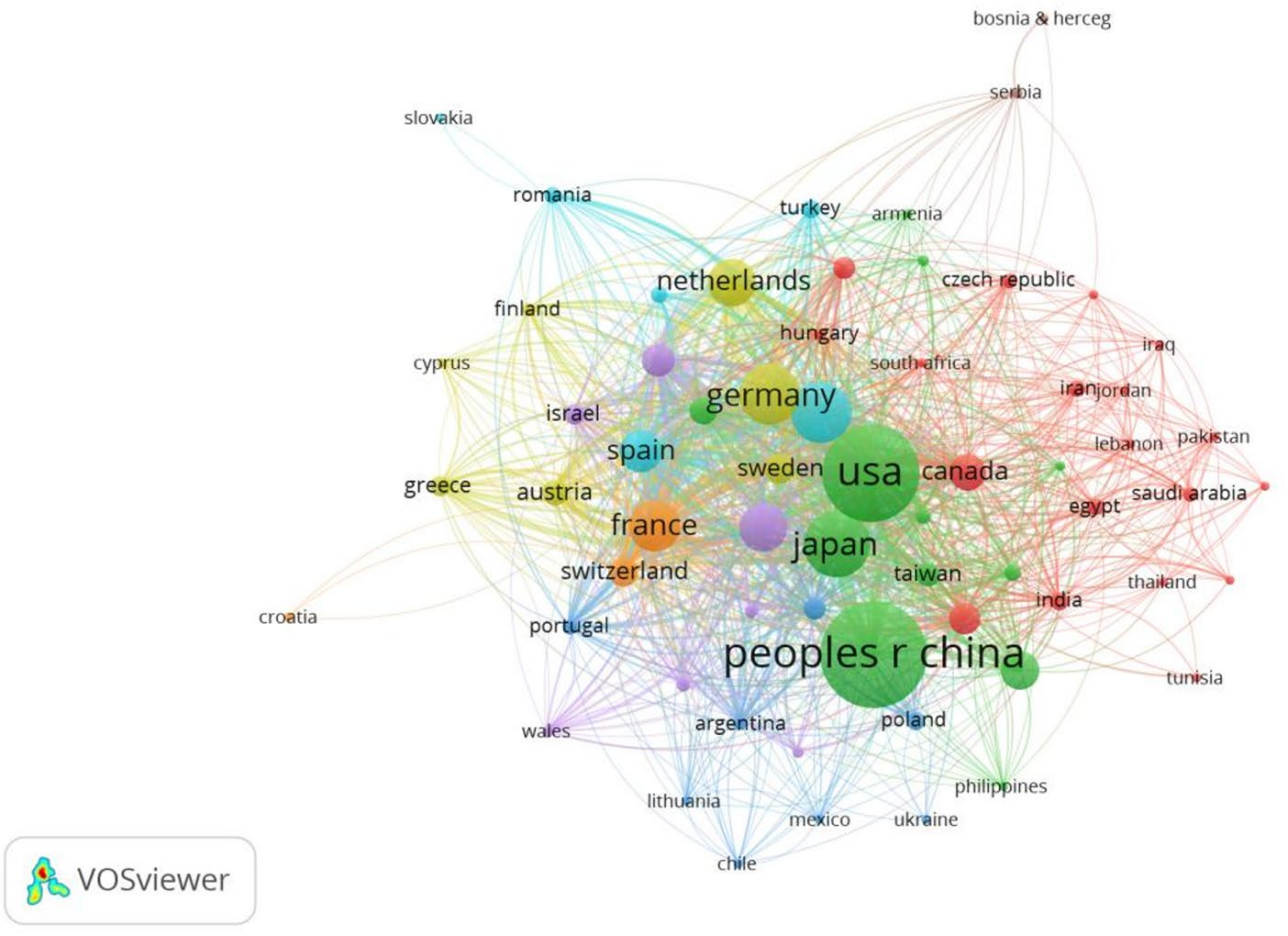
The co-occurrence of countries

To understand the national research contribution in the field of CCLM, we used VOSviewer to visualize and analyze publications from 108 countries involved in this field in the past decade, with results shown in Fig. 4. The larger nodes indicate more publications, the links represent the collaboration between authors, and the thicker the connecting line, the higher the number of collaborative communications between the two countries; different colors represent different clusters. It can be seen that the distribution of issuing countries in the field of CCLM is mainly concentrated in China, the United States, and Japan, and these three countries contribute more research in this field. We further analyzed the high-producing countries in this field, and Table 3 demonstrates the top five countries in terms of the number of publications. Chinese scholars contributed the most publications in this field, but publications by American scholars had the highest average number of citations, with 1974 publications receiving 67,565 citations.

**Table 3:** Top 5 countries in the field of CCLM

Rank	Country	Number of publications	Citations	Average citation/publication
1	China	2332	55,056	23.61
2	USA	1974	67,565	34.23
3	Japan	924	24,167	26.15
4	Germany	760	25,689	33.80
5	Italy	759	24,009	31.63

#### Bibliometric analysis of keywords

**Fig. 5:**
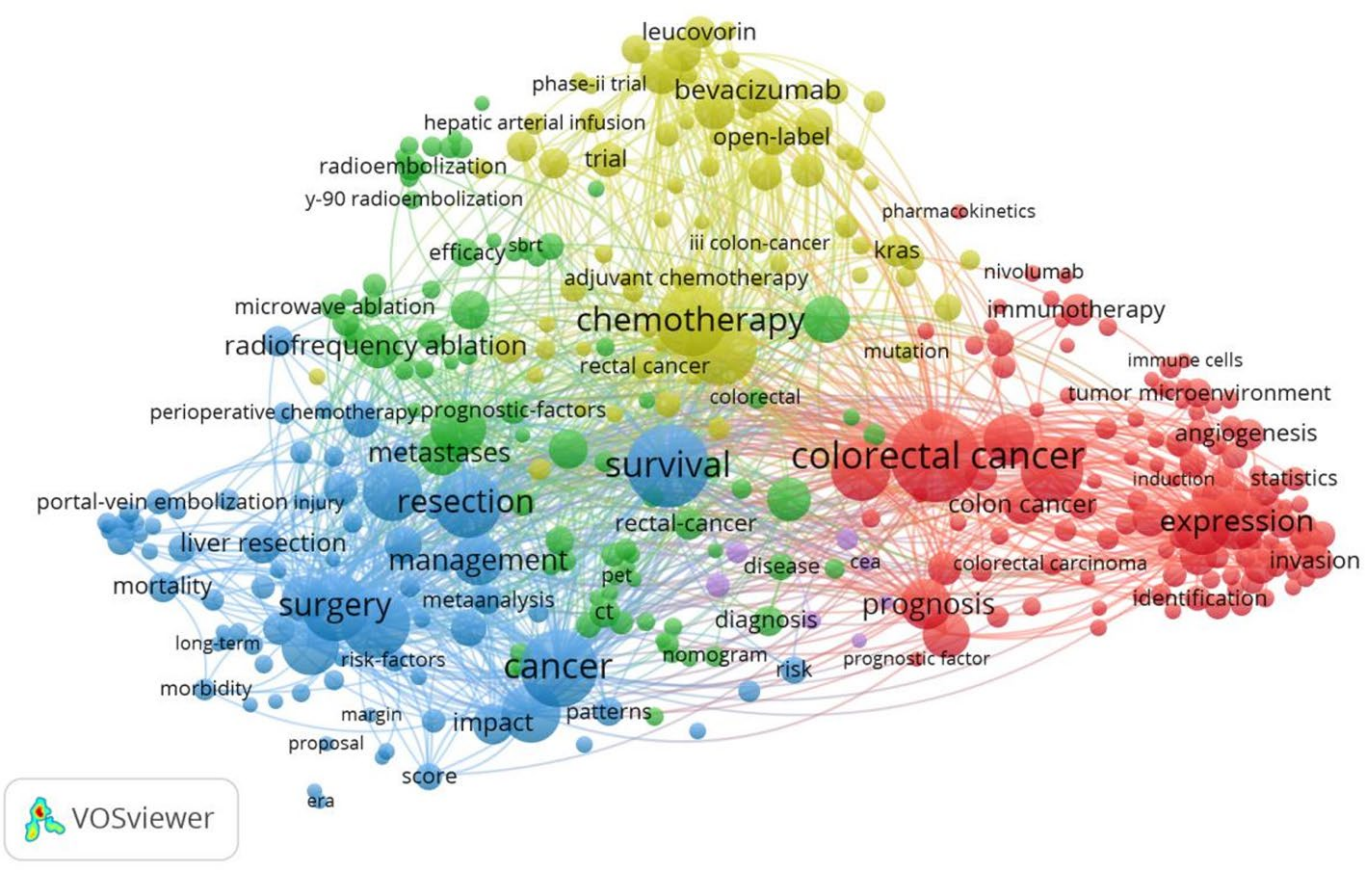
The co-occurrence of keywords

**Fig. 6:**
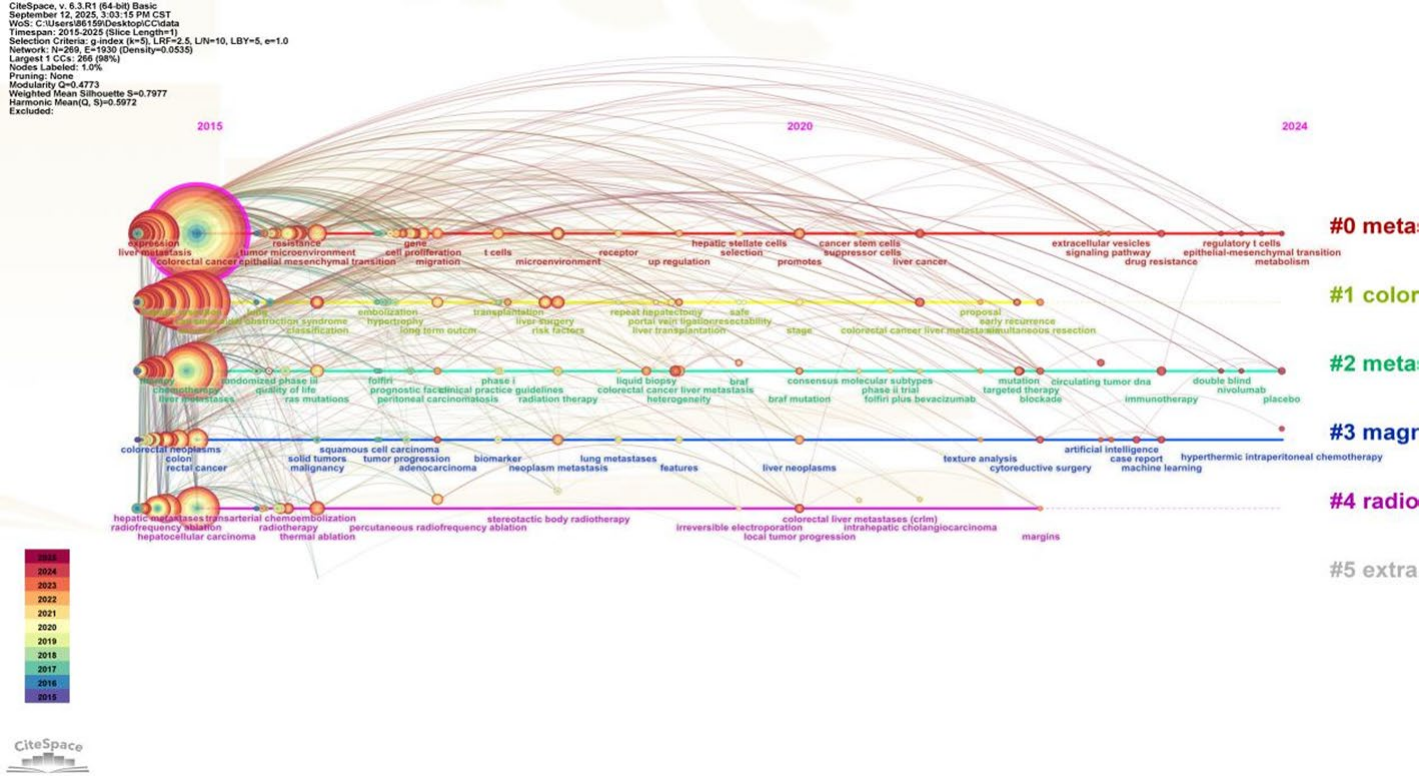
The clustering timeline view of keywords

Keywords condense the core and essence of literature, and keyword co-occurrence analysis can be used to identify research hotspots in the research field [28, 29]. We used VOSviewer to draw the co-occurrence network view of keywords for 8656 publications and selected 367 keywords with a frequency greater than 37 (including 37) for analysis based on Price’s Law, and Fig. 6 presents the visualization results [27]. In Fig. 5, the larger the round nodes, the more often the keywords appear, and the more representative of the hotspots in the field; the line between the nodes represents the strength of the association, and the thicker the line indicates that the two co-occur more often in the same literature; node color represents different clusters, research themes. To have a clearer understanding of the specifics of the keywords, the top 10 keywords in terms of frequency were made into Table 4, which shows that “colorectal cancer”, “survival”, “cancer”, “chemotherapy”, “liver metastases”, “hepatic resection”, “surgery”, “resection”, “liver metastasis”, “expression”, and other high-frequency keywords constitute the research hotspots in the field of CCLM, which have received the attention of a large number of scholars and produced corresponding research publications.

**Table 4:** Top 10 keywords in the field of CCLM

Rank	Keyword	Occurrences
1	Colorectal cancer	2331
2	Survival	1914
3	Cancer	1691
4	Chemotherapy	1383
5	Liver metastases	1375
6	Hepatic resection	1275
7	Surgery	1211
8	Resection	1189
9	Liver metastasis	980
10	Expression	906

#### Clustering timeline analysis of keywords

Keywords can be clustered and analyzed to derive the basic status of each research topic within that research territory. In order to clearly identify the inflection point of the discipline development and the temporal pattern of the frontier, the keyword co-occurrence graph can be arranged according to the time series, so as to show the distribution of the research hotspots in each time period [30]. In this study, we carried out the timeline graph with the help of Cilespace, selecting the node as Keyword, setting the Slice Length to 1 and the Selection Criteria to k = 5, and the results are shown in Fig. 6. The node size is proportional to the popularity of the keyword in the domain, the node color change from purple to red corresponds to the time change from 2015 to 2025, the connecting lines represent the interactions between the keywords, and the different clusters represent different research topics. As can be seen from Fig. 6, the high-frequency keywords “metastasis”, “colorectal liver metastases”, and so on are the core research questions; “Magnetic resonance”, “radiofrequency ablation”, and other words reflect that imaging and therapeutic technology are important research directions. The research on colorectal liver metastases has been on the rise in the last decade, with the early focus on basic research on metastatic mechanisms, the mid-term research expanding to the improvement of clinical therapeutic strategies, and the more recent research moving toward the frontiers of precision medicine and multidisciplinary collaboration.

#### Bursts analysis

**Fig. 7:**
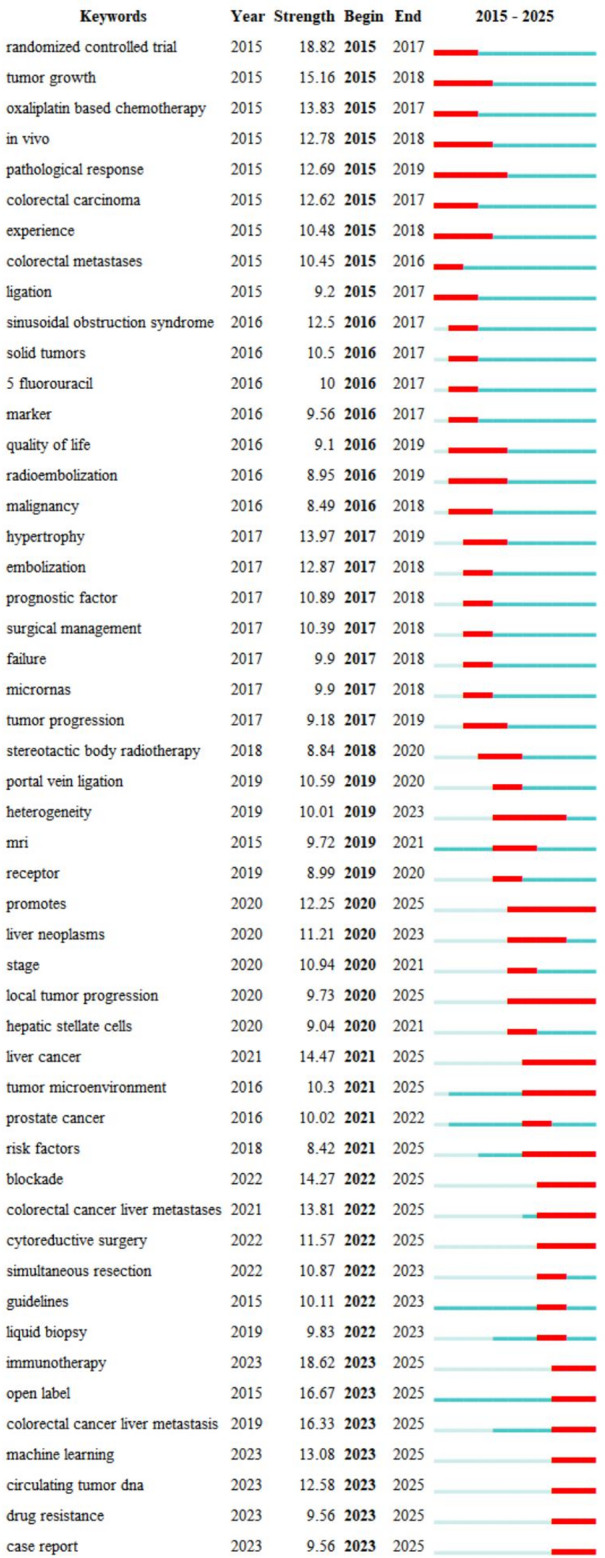
Top 50 keywords with the strongest citation bursts

In addition, we further analyzed the sudden outbreak of research hotspots in the field of CCLM by using the Bursts analysis function of CiteSpace, and a total of 108 outbreak words were found, of which we chose the top 50 outbreak words to be presented, and the results are shown in Fig. 7. It can be found that the eruption words in this field are more evenly distributed in time, mainly concentrated from 2015 to 2023. The strongest buzzword is “randomized controlled trial,” which appeared in 2015 and continued until 2017. “Promotes”, “local tumor progression”, “liver cancer”, “tumor microenvironment”, “risk factors”, “blockade”, “colorectal cancer liver metastases”, “cytoreductive surgery”, “immunotherapy”, “open label”, “colorectal cancer liver metastasis”, “machine learning”, “circulating tumor DNA”, “drug resistance”, and “case report” have all become recent research trends.

#### Analysis of cited journals

**Fig. 8:**
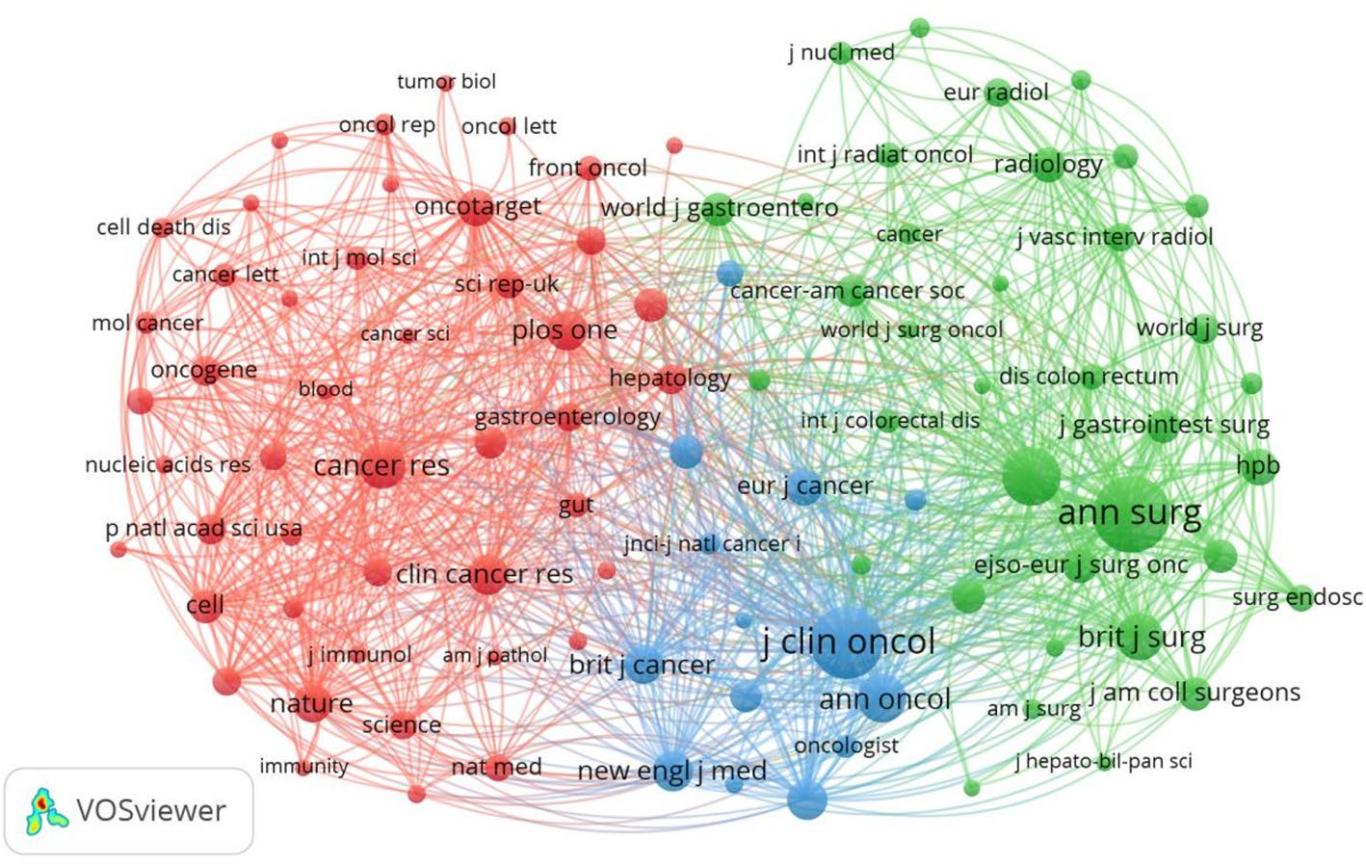
The co-citations of journals

In order to grasp the highly cited publications in the field of CCLM and the journals that published these publications, the co-citation mapping of journals was performed by VOSviewer, the threshold of the minimum number of co-citations of journals was set to 801, and 96 journals were retained for the co-citation analysis of the cited journals and the results are shown in Fig. 8. The 3 colors in the graph correspond to 3 different clusters. The top three journals with the highest number of citations were “Annals of Surgery”, “Journal of Clinical Oncology”, and “Annals of Surgical Oncology”, with corresponding citation counts of 17,357, 14,866, and 10,125, in that order.

#### Analysis of cited literature

**Fig. 9:**
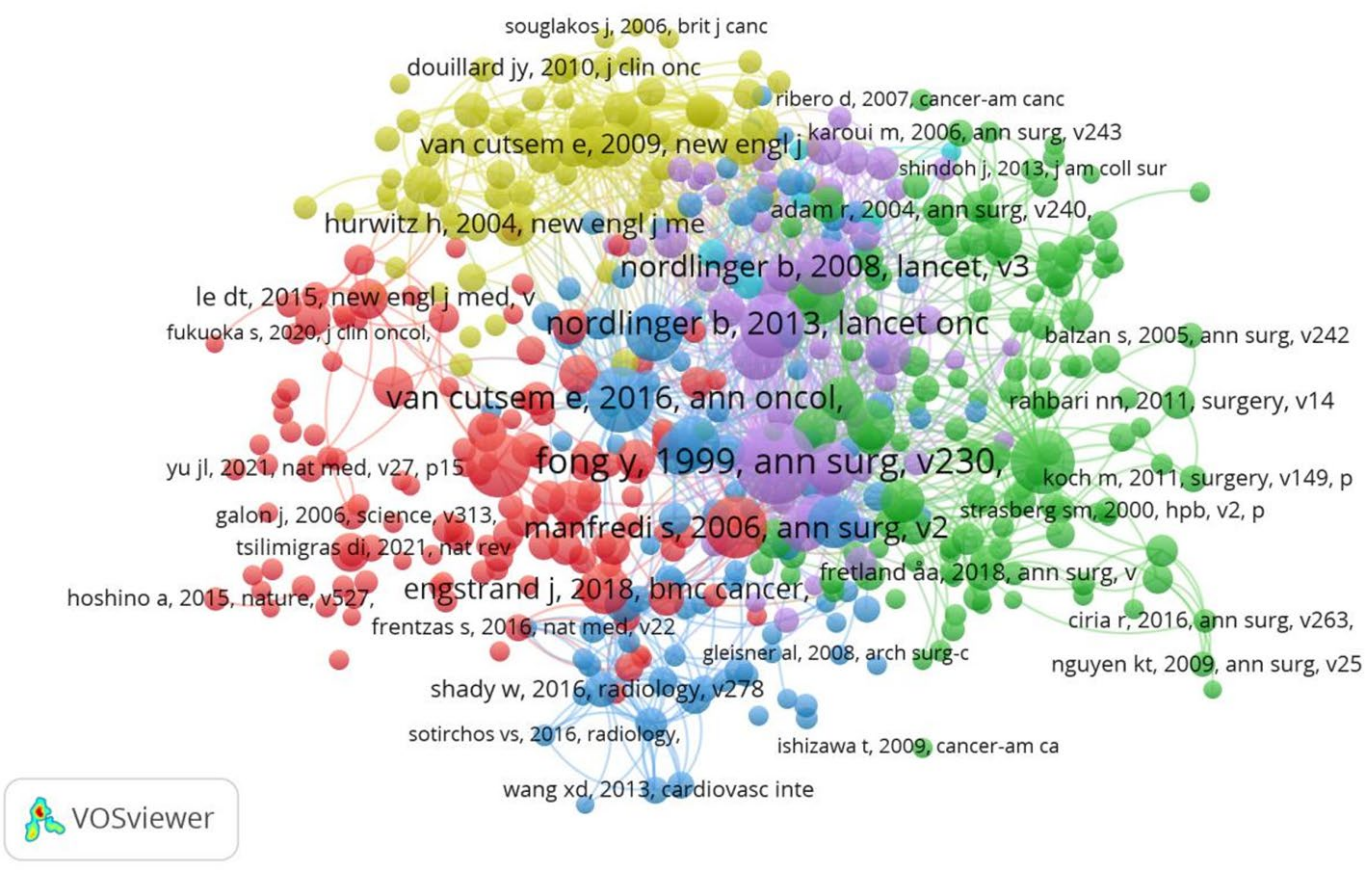
The co-citations of literature

In addition, we analyzed the co-citations of the literature, firstly using VOSviewer to analyze the top 10 cited literature in the field from 2015 to March 2025, as shown in Table 5. The most cited literature was “Clinical score for predicting recurrence after hepatic resection for metastatic colorectal cancer—Analysis of 1001 consecutive cases”, by Fong et al. [31] scholars, focuses on the analysis of 1001 cases to demonstrate that resection of hepatic colorectal metastases produces long-term survival and cure, and that long-term outcomes can be predicted from five criteria that are readily available to all patients considering resection. This is followed by literature by Van Cutsem et al. [32] with 682 citations and Nordlinger et al. [33] with 643 citations. Next, we set the threshold for the minimum number of co-citations in the literature to be 50, and 495 documents were retained for the co-citation analysis of the cited literature and the co-citation relationship is shown in Fig. 9. A node represents a reference, and the size of the node is positively correlated with the co-citation frequency of the reference. The larger the node, the more references it represents. The connecting lines between the nodes represent the strength of the association, and different colors indicate different clusters in the network visualization.

**Table 5:** Top 10 cited literature in the field of CCLM

Rank	Title	Year	Citations
1	Clinical score for predicting recurrence after hepatic resection for metastatic colorectal cancer—Analysis of 1001 consecutive cases [31]	1999	1043
2	ESMO consensus guidelines for the management of patients with metastatic colorectal cancer [32]	2016	682
3	Perioperative FOLFOX4 chemotherapy and surgery versus surgery alone for resectable liver metastases from colorectal cancer (EORTC 40983): long-term results of a randomised, controlled, phase 3 trial [33]	2013	643
4	Global cancer statistics 2020: GLOBOCAN estimates of incidence and mortality worldwide for 36 cancers in 185 countries [1]	2021	618
5	Classification of surgical complications—A new proposal with evaluation in a cohort of 6336 patients and results of a survey [92]	2004	615
6	Epidemiology and management of liver metastases from colorectal cancer [93]	2006	604
7	Perioperative chemotherapy with FOLFOX4 and surgery versus surgery alone for resectable liver metastases from colorectal cancer (EORTC Intergroup trial 40983): a randomised controlled trial [81]	2008	510
8	New response evaluation criteria in solid tumours: Revised RECIST guideline (version 1.1) [94]	2009	498
9	Improved Survival in Metastatic Colorectal Cancer Is Associated With Adoption of Hepatic Resection and Improved Chemotherapy [95]	2009	415
10	Actual 10-year survival after resection of colorectal liver Metastases defines cure [96]	2007	408

#### Analysis of citing and cited journals

**Fig. 10:**
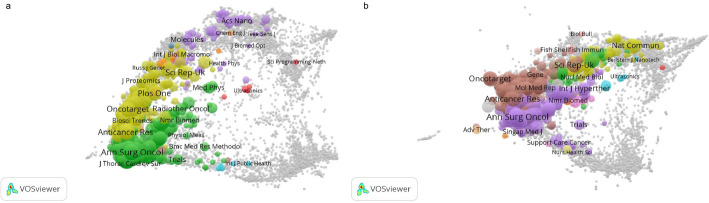
The bi-plot of citing and cited journals

The superimposed bi-plot analysis of the citing journals and the cited journals can clearly show the knowledge picture in the field of CCLM research, where the citing journals reflect the hotspots and cutting-edge directions of the current research, and the cited journals reveal the knowledge base and disciplinary sources of the research in this field [34]. Therefore, this study used VOSviewer to visualize the distribution of the citing journals and cited journals in the literature. It can be seen that the distribution of citing journals (Fig. 10A) and cited journals (Fig. 10B) is characterized by a clear distribution in terms of subject areas. The citing journals are mainly concentrated in the fields of oncology, surgery, imaging, etc. Journals such as “Oncotarget”, “Anticancer Research”, and “Annals of Surgical Oncology” are at the core of the network, which publishes a large number of high-quality and original research results, and play an important leading role in promoting the development of liver metastasis research in CRC. The cited journals also include some journals of other basic disciplines, which provide a solid foundation for the innovative development of the field, indicating that the research in the field of CCLM has drawn extensively on multidisciplinary theories and methods in the process of continuous deepening.

### Clinical progress analysis

**Fig. 11:**
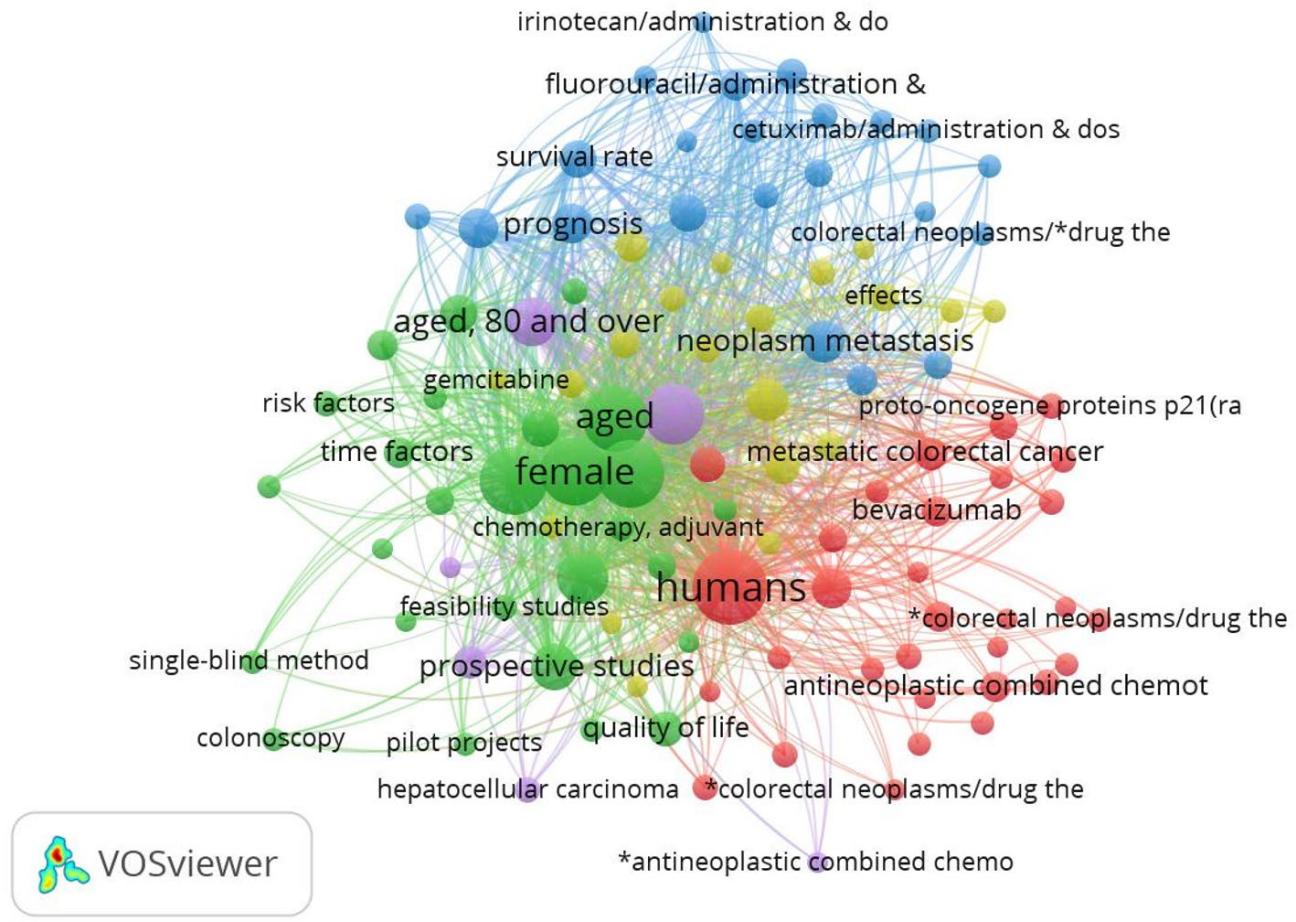
The co-occurrence of keywords for clinical trials

We also used VOSviewer to create a co-occurrence network view of keywords from 3220 clinical trial articles related to CCLM. Based on Price’s Law, we selected 106 keywords with a frequency greater than or equal to 43 for analysis. Figure 11 presents the visualized result, the larger the node in the figure, the higher the popularity of the keyword it represents. Among these, the top 10 keywords, as shown in Table 6, are “humans”, “female”, “male”, “middle aged”, “aged”, “adult”, “treatment outcome”, “aged, 80 and over”, “prospective studies”, and “disease-free survival”.

**Table 6:** Top 10 keywords for clinical trials

Rank	Keyword	Occurrences
1	Humans	3205
2	Female	2262
3	Male	2190
4	Middle aged	2119
5	Aged	2012
6	Adult	1487
7	Treatment outcome	884
8	Aged, 80 and over	742
9	Prospective studies	651
10	Disease-free survival	447

## Discussion

The field of CCLM has accumulated relatively rich literature resources. This study is based on VOSviewer and CiteSpace software to visualize and analyze the relevant research in the field of CCLM in the past ten years, systematically analyze the development of the research lineage in this field, and explore and analyze the development of the core authors, high-productivity countries, key journals, keyword clustering, the bi-plot of citing and cited journals, and clinical trial progress. The conclusions of the study based on econometric analysis are summarized as follows.

1. Over the past decade, the number of publications in the CCLM field has steadily increased, coinciding with a period of significant clinical advances.
2. This field has matured, establishing a relatively large number of core authors and their collaborative networks. These networks typically center around major cancer centers, leading clinical trials, and formulating treatment guidelines.
3. Most journals publishing literature in this field are in oncology and surgery, among which “Cancers”, “Annals of Surgical Oncology”, and “Frontiers in Oncology” are more core journals with higher quality.
4. China contributes the most publications in the field, but the publications by American scholars have the highest average number of citations per publication and are more recognized in the field. This may be related to historical accumulation, international collaboration networks, a higher number of publications in top journals, or leading pioneering research.
5. Co-occurrence analysis and evolution analysis of keywords revealed that the field has developed rapidly in terms of breadth and depth in the last decade, and the research involves all aspects of CCLM, such as mechanism, examination, treatment, and other basic and clinical aspects.
6. The co-citation analysis shows the journals and literature with high citation counts, providing scholars with a reference to better understand and grasp the research framework and vein of this field and explore potential research directions.
7. The superposition analysis of citing journals and cited journals shows that the vigorous and in-depth development of the research in this field involves the cross-fertilization of multiple disciplines, which provides a solid theoretical and research foundation for the innovative development of this field.
8. Clinical research on CCLM has mainly focused on prospective studies, with an emphasis on gender, age, treatment outcomes, and disease-free survival.

Keyword and burst word analysis can reveal research hotspots in the CCLM field and provide particularly insightful perspectives for analyzing clinical relevance. By analyzing the keywords and burst words in the field of CCLM in the last decade, we can find that keywords related to molecular mechanisms and screening appear more frequently from 2015 to 2025, such as “local tumor progression”, “tumor microenvironment”, “circulating tumor DNA”, “epithelial mesenchymal transition”, “platelet-derived endothelial cell growth factor”, “tumor-associated antigens”, “doppler perfusion index”, “stem cells”, “liquid biopsy”, “cell-free DNA”, “DNA methylation”, “microvessel density”, “tissue microarray”, “computed tomography”, “machine learning”, etc. This indicates that research into the molecular mechanisms of CCLM and early screening studies is necessary and has become a hot topic in the field. Additionally, research on the treatment and prognosis of CCLM has also received attention from scholars. “liver resection”, “cytoreductive surgery”, “immunotherapy”, “neoadjuvant systemic therapy”, “adjuvant chemotherapy”, “transarterial chemoembolization”, “radiofrequency ablation”, “microwave ablation”, “thermal ablation”, “antiangiogenic therapy”, “drug delivery”, “drug resistance”, “chemotherapy-induced liver injury”, “local recurrence”, and other keywords also appear with high frequency.

We further analyze and discuss the burst words in it, which continue to this day to explore the research hotspots and directions in the field of CCLM. First of all, risk factors associated with the development of CCLM, such as hyperlipidemia [35], chronic psychological stress [36], and pharmacologic liver injury [37], are also noteworthy and can assist in assessment and be used for health promotion. The diagnosis of CCLM mainly relies on imaging and puncture biopsy techniques, but there is no simple and fast early diagnostic method, so many scholars have shifted their attention to early prediction and diagnostic models, and many models based on machine learning techniques combined with conjunctival blood test indexes and diagnostic imaging have been developed [38–40]. By extracting patient information through machine learning algorithms, we can predict tumor biological behavior, treatment responses (such as liver damage after chemotherapy), and prognosis, enabling more precise decision-making [41, 42]. The outbreak of “machine learning” around 2023 marks the entry of CCLM research into a new paradigm of data-driven precision medicine. The timing of this explosion coincides with the enhancement of large databases by several institutions and the maturity of open-source algorithm libraries, allowing complex AI models to be widely applied in clinical research. The rapid development of Artificial Intelligence (AI) provides unprecedented opportunities for early detection, robust stratification, and therapeutic decision-making for CCLM, and its combination will become a future research trend [43].

The molecular mechanism of CCLM has always been a hot research topic in this field, and revealing its molecular mechanism is of great value for early diagnosis, personalized treatment, and new drug development. The assessment of RAS and BRAF gene mutations, combined with microsatellite instability (MSI) or dMMR status, has been incorporated into current clinical practice. Other promising molecular biomarkers, such as co-occurring mutations and circulating tumor DNA (ctDNA), are under active investigation [44]. CCLM may involve multiple genetic alterations. Ding et al. [45] identified APOBEC3G, CD133, LIPC, and S100P as genes mediating liver metastasis in CRC, which could be used to predict liver metastasis. Stremitzer et al. [46] showed that variants in genes such as RALBP1, PDGFB, and ANGPT2 may be associated with escape mechanisms. The outbreak of “circulating tumor DNA” marks the transition of liquid biopsy from a promising research concept to a validated clinical tool, coinciding with the publication of several milestone prospective studies.“Liquid biopsy” and “circulating tumor DNA”are associated with breakthroughs in next-generation sequencing (NGS) technology [47]. In addition, it also reflects the urgent need for clinical treatment decision support and active and increasingly personalized adoption as a dynamic biomarker for predicting recurrence, monitoring treatment response, and guiding adjuvant therapy decisions [48, 49]. Circulating tumor DNA (ctDNA) methylation profiles reflect tissue differentiation and malignant transformation and help identify CCLM. Furthermore, studies have shown that tumor-naive ctDNA detection can detect molecular residual lesions in patients with CRC after liver metastasis and predict postoperative recurrence [50–53].

Numerous studies have shown that various signaling pathways, such as AKT/mTOR [54, 55], JAK–STAT [56], PI3K/AKT [57], TGF-β [58–60], etc., mediate the development of liver metastasis in CRC, and the related genes and signaling pathways may suggest the prediction of liver metastasis and the target of treatment. In addition, the tumor microenvironment has garnered more academic attention, erupting in 2021 and continuing to this day. The sustained focus on the “tumor microenvironment” aligns with a broader understanding that the metastatic process is not only driven by the mutations of the tumor cells themselves but is orchestrated through complex interactions between tumor cells and the tumor microenvironment. The emergence of “tumor microenvironment” and “hepatic stellate cells” coincided with the widespread adoption of single-cell sequencing technology [61]. Kawada et al. [62] reviewed a large number of studies showing that the chemokine-chemokine receptor system promotes CCLM through a variety of complementary actions, including the promotion of cancer cell migration, invasion, survival, and angiogenesis as well as the recruitment of bone marrow-derived cells into the tumor microenvironment. Liu et al. [63] showed that a cytokine (TWEAK) secreted by Th17 immune cells in the immune microenvironment can promote EMT in CRC cells and drive liver metastasis of CRC. Intercellular interactions affect changes in the tumor microenvironment, and exosomes derived from cancer cells are thought to be important drivers of ecological niche formation prior to metastasis in distant organs. Wang et al. [64] showed that tumor-derived exosomes may promote CRC metastasis by recruiting CXCR4-expressing stromal cells to form a permissive metastatic microenvironment. Sun et al. [65] suggested that exosome ADAM17 is involved in the formation of a pre-metastatic ecological niche and can be used as a biomarker for distant metastasis in CRC. There are also a few buzzwords that come to mind, such as metabolic reprogramming, stem cells, and so on. Cancer cells are able to support their development through reprogramming of metabolic pathways such as glycolysis, glutaminolysis, oxidative phosphorylation, and lipid metabolism. Thrombopoietin (TPO)-induced metabolic reprogramming [66] and Aldolase B-Mediated Fructose Metabolism [67] have been demonstrated to drive CCLM. Stem cells are thought to be a source of CRC recurrence, metastasis, and drug resistance, and corresponding genes and biomarkers can help identify cancer stem cells to combat CRC metastasis and recurrence [68–71].

In addition, research related to the treatment of CCLM is a major hot topic, with the main topics being treatment strategies, survival prognosis, patient selection, and case reports. In terms of surgical treatment, a more comprehensive evaluation system based on biological behavior and anatomical features, hepatectomy (cytoreductive surgery) [72], minimally invasive laparoscopic surgery [73], and translational therapy [74] for initially unresectable patients combined with chemotherapy, targeted and local therapies (e.g., hepatic arterial infusion chemotherapy, HAIC) [75, 76] have attracted a great deal of attention from scholars. In addition, there are stratified strategies of targeted therapy combined with genetic testing, optimization of immunotherapy [77], and technological innovations of local therapy, such as microwave ablation (MWA), radiofrequency ablation (RFA), stereotactic radiotherapy (SBRT) [78], and selective internal radiation therapy (SIRT) [79]. The emergence and explosion of “immunotherapy” can be attributed to trials that have changed clinical practice, such as the publication of the groundbreaking KEYNOTE-177 trial [9], which established immune checkpoint inhibitors (ICIs) as the first-line standard treatment for specific molecular subtypes of mCRC (dMMR or MSI-H tumor patients). The current treatment of CCLM has shifted from single surgical resection to individualized comprehensive treatment under multidisciplinary collaboration (MDT), which should be developed more closely in the future through the integration of real-world data, precise staging, and technological innovation [80].

Clinical research on CCLM is still mainly prospective studies, focusing on the selection of treatment regimens, their efficacy and safety, and outcomes such as disease-free survival in patients. “Randomized controlled trials” erupted between 2015 and 2017, marking the official transition of the CCLM field from the era of “empirical medicine” and “retrospective studies” to the era of “evidence-based medicine” and “precision medicine.” This eruption stemmed from the publication of long-term follow-up results of several key randomized controlled trials and the resulting widespread discussion and transformations in clinical practice, such as the landmark EORTC 40,983 trial, which published preliminary results as early as 2008 [81] and long-term follow-up results in 2013 [33], by around 2015, these long-term data had been widely discussed, accepted, and cited. Large-scale clinical trials translate fundamental and technological advances into conclusive clinical evidence, ultimately transforming and guiding clinical guidelines and practice. A multicenter, randomized trial by Avallone et al. [82] demonstrated that intermittent treatment after an induction phase of the first-line schedule of FOLFIRI plus panitumumab (PAN) improves safety and compliance with treatment in patients with unresectable RAS and BRAF wild-type metastatic colorectal cancer (mCRC) patients. Research by van et al. [83] showed the potential non-inferiority of thermal ablation compared with surgical resection in patients with small-size resectable colorectal liver metastases. Adam et al. [84] showed that, compared with chemotherapy alone, liver transplantation combined with organ allocation priority chemotherapy significantly improved the survival rate of patients with unresectable colorectal liver metastases.

## Future directions and challenges

Beyond delineating the current research status, our analysis helps identify potential gaps and future directions. In the future, basic and clinical research on the mechanism, examination, and treatment of CCLM will be carried out continuously.

Among these, we believe the tumor microenvironment (TME) has been and will remain an absolute hotspot in cancer research, requiring substantial basic and clinical translational studies in the future, such as novel therapies that disrupt the metastatic niche [85, 86]. Early screening and prediction of CCLM occurrence and recurrence risk remain areas requiring further research. Strong signals from ctDNA indicate the need for further prospective studies to validate its utility not only for recurrence monitoring but also for real-time guidance on the duration of neoadjuvant and adjuvant therapies, as well as for the early detection of micrometastatic disease [87]. Machine learning can integrate multi-omics data (genomics, radiomics, pathology, etc.) through artificial intelligence algorithms to create highly personalized predictive models for treatment response, recurrence risk, and optimal therapeutic strategies [88]. Substantial prospective studies remain necessary to progressively realize AI-driven precision medicine.

Furthermore, future research should further integrate multidisciplinary resources, emphasize clinical translation, explore the potential applications of new technologies, and advance CCLM research to greater depths. This will provide stronger support for clinical treatment and improve patient outcomes.

## Limitations

Our study provides valuable insights into the research trends and hotspots in the field of CCLM in English-language core journals, which can help more scholars to have a comprehensive understanding of the development of the field and facilitate their exploration of potential research directions. It should be noted that bibliometrics captures “academic impact” (publications and citations), which is not equivalent to “intrinsic quality” or “direct clinical value”. Highly cited works may be groundbreaking, controversial, or methodologically flawed. We encourage readers to consider other forms of evidence (such as systematic reviews and clinical guidelines) to make a comprehensive assessment of progress in the field.

Of course, there are some limitations in this study. For example, only English-language literature in WoSCC and PubMed was included to ensure the quality and consistency of the collected data, which may lead to the problem that the study is not comprehensive enough. This selection approach risks overlooking significant publications from other databases such as Scopus, Embase, or regional databases, as well as high-quality research published in languages other than English in native-language journals (e.g., Chinese, Japanese, Spanish) [89–91]. This may introduce language bias and compromises the comprehensive representation of research contributions from non-English-speaking regions, particularly when describing national productivity and collaborative networks or regional clinical practices, thus affecting the integrity of the global research landscapeand. In the citations, self-citations were not adjusted, which may affect the objectivity of the results.

Future bibliometric studies may benefit from including multilingual databases such as CNKI or Embase to provide a more comprehensive global perspective. Also, bibliometric methods focus on quantitative output and may not fully reflect research quality or content depth. Differences in software versions and parameters and the inevitable subjectivity in analyzing and interpreting the data, can lead to slight variances in analyses.

## Conclusion

In conclusion, this study used bibliometric methods to analyze, visualize, and summarize the relevant publications on CCLM from 2015 to 2025, and systematically sorted out the current research status, trends, and hotspots in this field, which can provide valuable guidance for the researchers to further understand the current research status and to grasp the potential research directions in this field in the future. The field of CCLM still has ambitious research prospects and the significance and value of further deepening the research.

## Data Availability

The datasets generated during and/or analysed during the current study are available from the corresponding author on reasonable request.
